# Effects of Internalized Gold Nanoparticles with Respect to Cytotoxicity and Invasion Activity in Lung Cancer Cells

**DOI:** 10.1371/journal.pone.0099175

**Published:** 2014-06-05

**Authors:** Zhengxia Liu, Yucheng Wu, Zhirui Guo, Ying Liu, Yujie Shen, Ping Zhou, Xiang Lu

**Affiliations:** Department of Geriatrics, the Second Affiliated Hospital, Nanjing Medical University, Jiangsu, China; Northwestern University, United States of America

## Abstract

The effect of gold nanoparticles on lung cancer cells is not yet clear. In this study, we investigated the cytotoxicity and cell invasion activity of lung cancer cells after treatment with gold nanoparticles and showed that small gold nanoparticles can be endocytosed by lung cancer cells and that they facilitate cell invasion. The growth of A549 cells was inhibited after treatment with 5-nm gold nanoparticles, but cell invasion increased. Endocytosed gold nanoparticles (size, 10 nm) notably promoted the invasion activity of 95D cells. All these effects of gold nanoparticles were not seen after treatment with larger particles (20 and 40 nm). The enhanced invasion activity may be associated with the increased expression of matrix metalloproteinase 9 and intercellular adhesion molecule-1. In this study, we obtained evidence for the effect of gold nanoparticles on lung cancer cell invasion activity in vitro. Moreover, matrix metalloproteinase 9 and intercellular adhesion molecule-1, key modulators of cell invasion, were found to be regulated by gold nanoparticles. These data also demonstrate that the responses of the A549 and 95D cells to gold nanoparticles have a remarkable relationship with their unique size-dependent physiochemical properties. Therefore, this study provides a new perspective for cell biology research in nanomedicine.

## Introduction

Previous studies identified that gold nanoparticles (Au-NPs) show little cytotoxicity despite their efficient uptake into human cells by endocytosis [1], [2], making them suitable candidates for nanomedicine. Besides their biocompatibility, the fact that they are easy to synthesize, characterize, and surface modify contributed to attract much attention in various biomedical applications. Au-NPs have been investigated as drug delivery vehicles and photothermal therapy and molecular imaging tools for potential biodiagnosis [3], [4]. Nanoparticle-based therapeutic strategies for cancer treatment are mainly based on the delivery of chemotherapeutic agents to induce apoptosis [5]. The primary reasons for using nanoparticles as carriers for therapeutic delivery are to enable multimodal functionalities, such as imaging or specific targeting, to increase tissue permeability and site-specific drug accumulation, and to reduce side effects to healthy tissues [6]. Currently, Au-NPs are used in different biomedical applications: not only can they be used as scaffolds for increasingly potent cancer drug delivery but they can also serve as transfection agents for selective gene therapy and as intrinsic antineoplastic agents[7]–[9]. Dreaden et al. [8] have shown that targeted Au-NPs are capable of altering the cell cycle, including cell division, signaling, and proliferation.

Despite the widespread application of Au-NPs, a clear understanding of how biological systems respond to the nanoparticles is vital, and characterization of the unique size-dependent physicochemical properties of the Au-NPs is a critical component. A previous study proved that the surface size of Au-NPs plays a large role in their therapeutic effect [10]. Au-NPs of very small diameter (<2 nm) can penetrate cells and cellular compartments such as the nucleus and be extremely toxic [11]. For example, it was found that spherical Au-NPs with a diameter of 1.4 nm induce necrosis and mitochondrial damage in various cell lines via oxidative stress mechanisms, which may be associated with their well-known catalytic activity at that size [12]. A recent study by Connor et al. [1] reported that significant amounts of larger Au-NPs (e.g., 18 nm in diameter) penetrate into cells, but that these Au-NPs are not inherently toxic to human cells. Chithrani et al. [13] studied the relationship between Au-NPs and HeLa cells and suggested that Au-NPs entered the cells via receptor-mediated endocytosis at a threshold size of approximately 50 nm. Since there are no safety regulations yet, the effect of Au-NPs on cells still requires further study.

Invasion and metastasis are important pathologic features of cancer cells. Invasive capacity is the single most important trait that distinguishes benign from malignant lesions [14]. Indeed, invasive tumor cells can escape surgical resection and be responsible for tumor recurrence. Despite advances in surgery, chemotherapy, and radiotherapy, relapse is almost inevitable in the presence of an aggressive metastatic spread [15], [16]. The process of invasion and metastasis includes cell proliferation, dissociation from the primary lesions, degradation, and permeation into the extracellular matrix (ECM), migration in the blood or lymph stream, adhesion and growth in a secondary organ [17]. Previous reports have described that intercellular adhesion molecule-1 (ICAM-1) and matrix metalloproteinase 9 (MMP-9) are involved in cancer cell adhesion, invasion, and migration, which contribute to cancer metastasis [18]. These factors have been considered as prognostic biomarkers for lung cancer progression [19]. Further studies on the effects of Au-NPs on the expression of these proteins are needed.

Since cytotoxicity may not be the only effect that nanoparticles can induce, in this study, we focused on cell proliferation, invasion activity, and protein expression, all of which may be affected by the presence of Au-NPs. To investigate the importance of particle size in nanomedicine, we chose four different Au-NPs sizes (i.e., 5 nm, 10 nm, 20 nm, 40 nm) for an in-depth analysis of this system.

Finally, we chose to study these effects on two human lung cancer cell lines (i.e., A549 and 95D) because lung cancer is a major malignant tumor in China and has increasing incidence in most cities. In 2010, there were approximately 600,000 new cases of lung cancer in China [20]. The rates of morbidity continue to rise rapidly because of the serious air pollution [21]; however, knowledge of the biological interactions and responses of Au-NPs with human lung cancer cells is very limited. Moreover, to gain a fundamental understanding of the processes involved, we felt that it was important to initially focus on the effects of nanoparticles on individual cancer cells, rather than on entire organs, where other factors can complicate the interpretation of the results. Therefore, the goal of this research was to provide an important basis for applying Au-NPs in lung cancer therapy.

## Methods

### Preparation and Characterization of Citrate-Capped Au-NPs

Au-NPs (5 nm and 10 nm) were synthesized using a tannic acid/citrate solution, in which tannic acid plays the role of a reducing agent and citrate acts as a stabilizing agent [22]. For each synthesis, two initial solutions were required: (a) 1 mL of 1% (w/v) HAuCl_4_ solution, which was added to 79 mL of water; and (b) a mixture consisting of 4 mL of 1% (w/v) citrate solution, 0.7 mL or 0.1 mL of 1% tannic acid, and water (to achieve a final volume of 20 mL). Both (a) and (b) solutions were heated to 60°C in water bath and then solution (b) was added to (a) under constant stirring. The resulting Au-NPs were cooled to room temperature (RT) for subsequent experiments.

Syntheses of 20-nm and 40-nm Au-NPs stabilized by citrate ions were conducted using the classic citrate reduction method [23]. For each synthesis, 100 mL of 0.01% HAuCl_4_ solution was heated to boiling. Selected volumes (4.5 mL or 1.0 mL) of 1% citrate solution were added and boiling was continued until the color of the solution turned into ruby red. The citrate-capped Au-NPs solution was cooled naturally to RT.

The morphology of the Au-NPs was observed by using transmission electron microscopy (TEM; JEM-2100EX, JEOL, Tokyo, Japan) at accelerating voltage of 200 kV. Ultraviolet-visible (UV-Vis) spectra were acquired with a Shimadzu UV-3600 spectrophotometer in the range of 300–1100 nm.

### Cell Culture

A549 and 95D cells (Shanghai Institutes for Biological Sciences, Chinese Academy of Sciences) were cultured in Dulbecco’s Modified Eagle’s Medium (DMEM) supplemented with 10% (v/v) fetal bovine serum (FBS), 100 units/mL penicillin, and 100 µg/mL streptomycin (all from GIBCO, Invitrogen) at 37°C in a humidified atmosphere containing 5% CO_2_. Cells were pre-cultured in the medium overnight and then treated either with or without 50 µg/mL Au-NPs solution for an additional 48 h. Cells were harvested by trypsin-ethylenediamintetraacetic acid (EDTA; GIBCO, Invitrogen) detachment at the logarithmic growth phase and centrifuged. The cells were then resuspended in DMEM with 10% FBS for subculture or other uses.

### Uptake and TEM Studies

The uptake of Au-NPs was also examined using TEM. Before exposure to the Au-NPs, the A549 cells and 95D cells were plated at a concentration of 1×10^6^ cells per dish on a 100-mm culture dish (corning, USA) containing the growth medium and incubated at 37°C with 5% CO_2_ for 24 h. The Au-NPs were then added. After exposure to Au-NPs for 48 h, the cells were fixed in 3.7% (v/v) paraformaldehyde in phosphate-buffered saline (PBS) for 20 min at RT. Subsequently, cells were prepared for TEM analysis as follows: cells were fixed in 1% (w/v) osmium tetroxide for 2 h, dehydrated in a graded series of 30%, 50%, 70%, 80%, and 90% ethanol, and treated three times with 100% ethanol for 15 min each. The samples were then embedded in a mixture of resin in propylene oxide polymerized at 80°C. Ultrathin sections for TEM were prepared using a diamond knife and the samples were analyzed using a transmission electron microscope.

### Cell Viability Assay

A549 and 95D cells were seeded in 96-well plates at a low confluence (2000 cells/well), allowed to attach overnight, and treated with Au-NPs (control, 5 nm, 10 nm, 20 nm, and 40 nm). After 24 h, 48 h, and 72 h, a cell viability assay reagent (Cell counting kit-8, Kaiji, Nanjing) was added to each well and incubated for 1, 2, 3, and 4 h, respectively. Absorbance values at 450 nm were recorded using a microculture plate reader (BioRad) and the cell viability expressed as a percentage of the untreated control (100% cell viability). The effect of the Au-NPs with different sizes on the viability of the cells was measured in triplicate, and the experiments were repeated at least thrice.

### Apoptosis Detection by Annexin V/Propidium Iodide (PI) Staining

Cells in the log phase were seeded onto a 6-well culture plate at a density of 1×10^5^ cells per well, incubated at 37°C in a CO_2_ incubator, and allowed to attach overnight. After treatment with Au-NPs (control, 5 nm, 10 nm, 20 nm, and 40 nm) for 48 h, apoptosis and necrosis were analyzed with the Annexin V-PI (BD Biosciences) apoptosis detection kit following the manufacturer’s instructions. The samples were analyzed using a BD FACS CantoII instrument (BD Biosciences).

### Flow Cytometry Analysis of the Cell Cycle

The cells were harvested using 0.25% trypsin with 1 mM EDTA solution and fixed for 12 h in 70% ethanol at 4°C. The fixed cells were then centrifuged at 3,000 rpm for 15 min to remove the ethanol thoroughly. The cells were then washed twice with 3 mL of PBS, resuspended in 1 mL of PI staining solution, and incubated for 15 min at RT. The staining solution consisted of 20 µg/mL PI and 0.2 mg/mL RNase A in PBS. The samples were subsequently analyzed using a BD FACS CantoII instrument (BD Biosciences). Twenty thousand events were collected from each sample. The percentages of cells in the G0/G1, S, and G2/M phases of the cell cycle were determined using the ModFit software (BD Biosciences).

### Invasion Assay

For the invasion assay, hanging cell culture inserts (8.0-µm pore size) were pre-coated with 50 µg/mL Matrigel (BD Biosciences) on the upper surface. Cells were pre-cultured in the medium overnight and then treated either with or without 50 µg/mL Au-NPs solution for additional 48 h, then cells were harvested and 2.5×10^5^ cells were plated in 200 µL DMEM supplemented with 0.2% bovine serum albumin (BSA) in the top of the chamber. The bottom of the well was added with 750 µL of DMEM containing 5% FBS. The invasion assay was carried out for 48 h in the cell culture incubator.

The cells were fixed by replacing the culture medium in the bottom and top of the chamber with 4% formaldehyde dissolved in PBS. After fixing for 15 min at RT, the chambers were rinsed in PBS and stained with 0.2% crystal violet for 10 min. After washing the chambers five times by dipping them in a large beaker filled with dH_2_O, the cells (now blue in color) at the top of the Matrigel membrane were removed by using several Q-tips. Cells were removed until no more blue dye could be removed with the Q-tips. Then, the cells that remained were those that had invaded and had reached the bottom of the membrane.

We also quantified the invasion cells using the QCM™ 24-well Cell Invasion Fluorometric Assay (Millipore) with ECMatrix-coated inserts, according to the manufacturer’s instructions. This assay provides an efficient system for quantitative evaluation of the invasion of tumor cells through a basement membrane model. A549 and 95D cells were grown for 2 d in complete medium, either in the absence (control) or in the presence of Au-NPs (5 nm, 10 nm, 20 nm, 40 nm). At the end of the treatment, cells were suspended in serum-free medium and seeded (2.5×10^5^ cells/250 µL) in each insert of a multiwall plate chamber. Serum (10%) was added to the serum-free medium in the lower chamber as chemoattractant. After a 48-h incubation period at 37°C, non-invading cells were removed from the top of the inserts. Next, the inserts were placed into a clean well containing a pre-warmed cell detachment solution and incubated at 37°C for 30 min. The inserts were removed from the wells, and lysis buffer/dye solution was added to the medium containing the detached cells for 15 min at RT. The mixtures were then read with a fluorescence plate reader (Bio-Tek, Synergy HT) using a 480/520-nm filter set. Fluorescence measurements were reported as relative fluorescence unit (RFU) values.

### Quantitative Reverse Transcription Polymerase Chain Reaction (qRT-PCR) Assays

Total RNA was isolated from untreated and Au-NPs–treated A549 and 95D cells using Trizol Reagent (Invitrogen Life Technology). Reverse transcription (RT) reaction was performed using 1 µg of total RNA, which was reverse transcribed into cDNA using oligo dT primer, and then qRT- PCR was carried out using the SYBR Green Mix (Applied Bio-systems). The primer sequences used for PCR were as follows: ICAM-1, forward ACACTAGGCCACGCATCTGAT, reverse AGCATACCCAATAGGCAGCAA; MMP-9, forward GGCTACGTGACCTATGACATCCT, reverse TCCTCCCTTTCCTCCAGAACA; glyceraldehyde 3-phosphate dehydrogenase (GAPDH), forward GGAGCCAAACGGGTCATCATCTC, reverse GAGGGGCCATCCACAGTCTTCT. PCR was carried out at 95°C for 30 s, at 60°C for 30 s, and 1 min at 70°C for 35 cycles. The comparative Ct method was used to calculate the relative abundance of the mRNA and the results for target gene expression were compared with those for GAPDH expression. The results were obtained from three independent experiments.

### Protein Quantitative Assays

MMP9 protein expression in the supernatant and cell lysate was measured using an MMP Panel 2 magnetic bead kit based on the Luminex technology. Briefly, MMP9 capture antibodies (Millipore) were conjugated to Luminex beads (beads region 33). MMP9 detection antibodies (Millipore) were conjugated to biotin through custom service provided by Millipore. Cells were lysed in MILLIPLEX MAP lysis buffer (Millipore) and diluted with equal volume of MILLIPLEX MAP cell assay buffer (Millipore). MMP9 capture antibody beads were diluted in 25 µL of MILLIPLEX MAP cell assay buffer and added to a magnetic plate (Millipore). Then, 25 µL of the diluted cell lysate or cell culture supernatant was transferred to each well of the solid plate and incubated for 2 h at RT with shaking. After the incubation, beads were washed twice with wash buffer, and 25 µL of detection antibodies was added into each well and incubated for 1 h at RT with shaking. After that, 25 µL of MILLIPLEX MAP streptavidin-phycoerythrin (Millipore) was added and incubated for 30 min at RT with shaking. Finally, sheath fluid was added after washing, and the signal was read using a Luminex FLEXMAP 3D™.

### Western Blot Analysis

For western blot analysis, A549 and 95D cells were plated on culture flasks and treated with Au-NPs (control, 5 nm, 10 nm, 20 nm, and 40 nm). After 48 h, 5–10×10^6^ cells were harvested and lysed with ice-cold lysis buffer. Equal amounts of protein were separated by sodium dodecyl sulfate-polyacrylamide gel electrophoresis (SDS-PAGE) and electrophoretically transferred onto polyvinylidene fluoride membranes. Membranes were blocked with 5% non-fat dry milk for 2 h and incubated overnight at 4°C with rabbit anti-MMP-9 antibody (1∶1000, Abcam), rabbit anti-ICAM-1 antibody (1∶500, Cell Signaling), or mouse anti-GAPDH antibody (1∶10000, Abmart). GAPDH was used as the housekeeping gene control and the expression levels of the MMP9 and ICAM-1 were normalized with respect to GAPDH. The proteins were detected with horseradish peroxidase-conjugated anti-rabbit or anti-mouse secondary antibodies and visualized with chemiluminescence reagents provided with the ECL kit (BioRad, USA). Immunoreactive bands were detected by enhanced chemiluminescence and quantified using a ChemiDoc XRS molecular imager (BioRad).

### Statistical Analysis

GraphPad Prism 5 statistical analyses software was used in all statistical analyses performed in this study (GraphPad Prism version 5.00 for Windows, GraphPad Software, San Diego California, USA). All results have been presented as mean ± standard deviation (SD). Statistical comparisons were conducted using one-way analysis of variance (ANOVA), followed by the Dunnett’s *t*-test for comparison with the control group. Differences were considered significant at P<0.05.

## Results

### Synthesis and Characterization of Au-NPs

**Figure 1:**
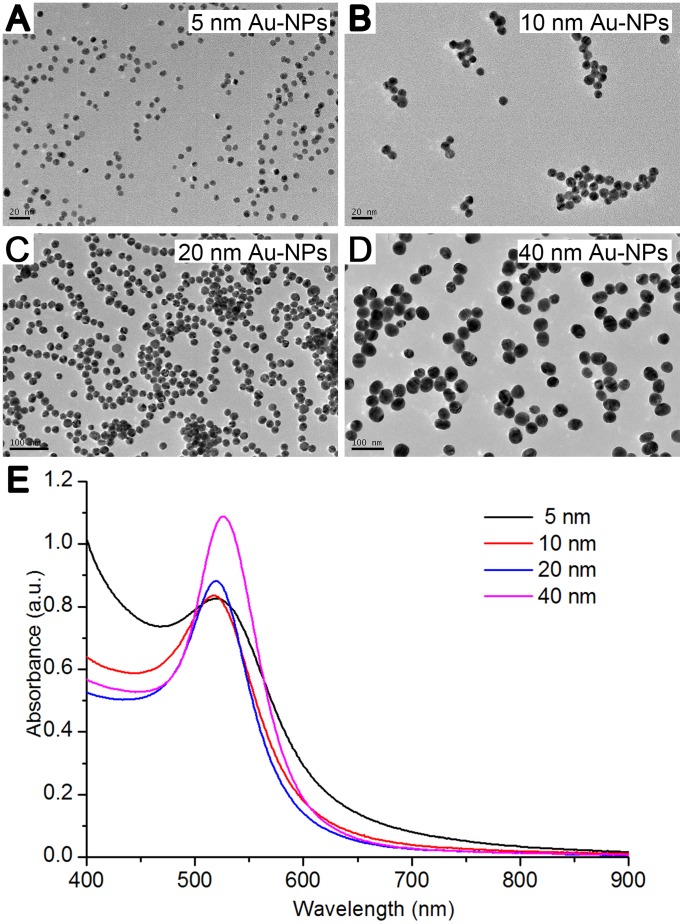
Characterization of the differently sized Au-NPs. Transmission electron microscopy (TEM) images of Au-NPs with diameters of (A) 5 nm, (B) 10 nm, (C) 20 nm, or (D) 40 nm. The insets show high-resolution images. Scale bars, 20 nm and 100 nm (as marked). E: UV-vis spectra of the Au-NPs with 5-nm, 10-nm, 20-nm, and 40-nm diameters.

Au-NPs synthesized with the citrate reduction method without any further modification were used for all the studies and are here referred to as unmodified nanoparticles. To determine the relationship between the size and the biological function of the nanoparticles, we used Au-NPs of four different sizes (5, 10, 20, or 40 nm) and characterized them by TEM and UV-Vis spectra (Figure 1). The particles were shown to be all spheres with narrow size distributions. The high electron densities of Au-NPs as well as the homogeneity of their shape and size made them highly evident under the transmission electron microscope.

### Internalization of Au-NPs

**Figure 2:**
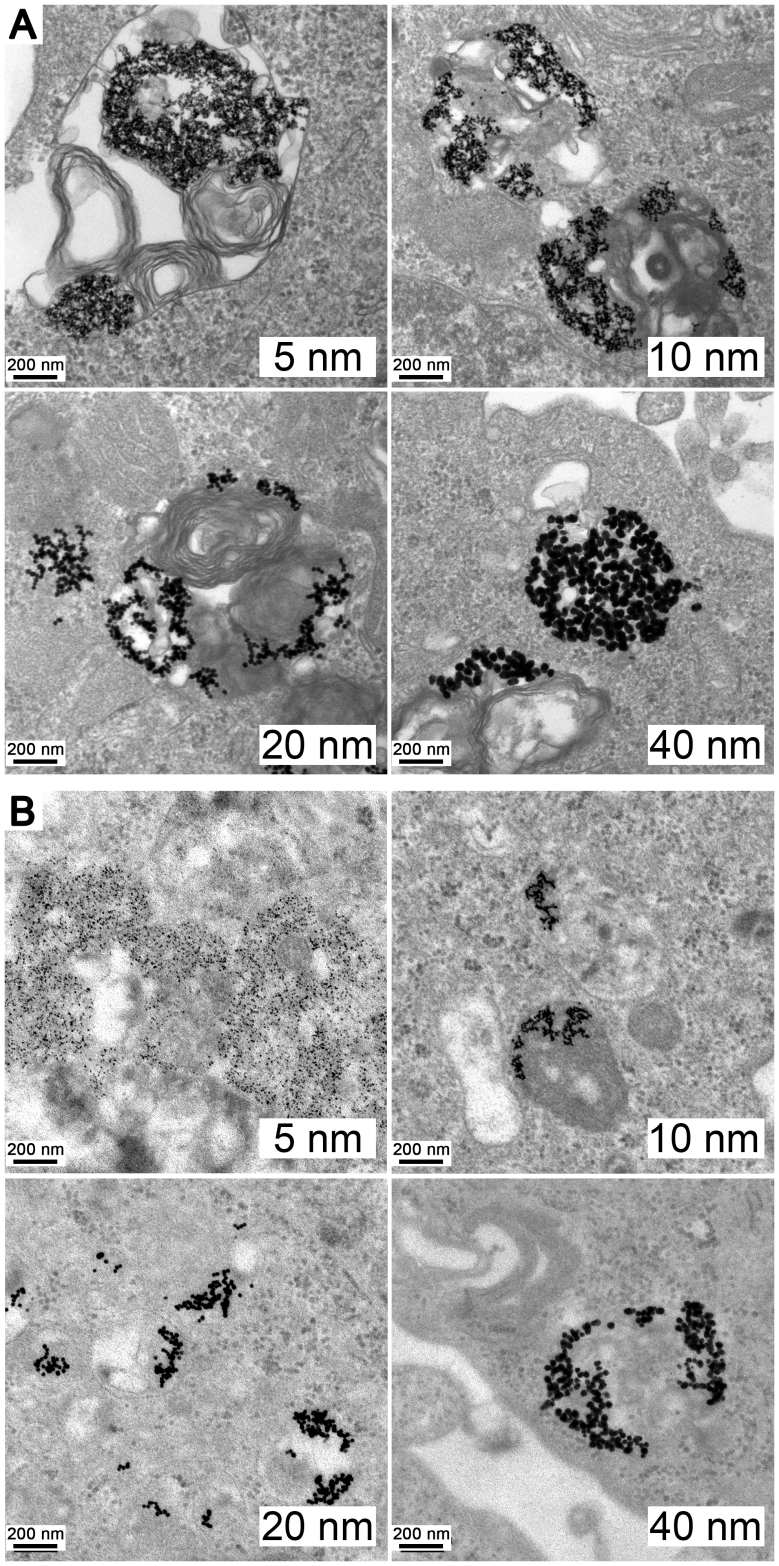
Transmission electron microscopy (TEM) images of Au-NPs trapped inside the cells. TEM images at 200-nm magnification showing the internalization of 25 µg/mL Au-NPs with various sizes (5 nm, 10 nm, 20 nm, and 40 nm) into (A) A549 and (B) 95D cells after 48 h of treatment.

To investigate whether Au-NPs with various sizes crossed the cell membrane and where they located, A549 and 95D cells were incubated for 48 h in the presence of Au-NPs (5, 10, 20, and 40 nm) in complete cell culture medium. Figure 2 shows the internalization of the differently sized Au-NPs. Most of the particles were found in membrane-bound vesicles or in the perinuclear region within the cells.

### Measurement of the Cytotoxicity of Au-NPs

**Figure 3:**
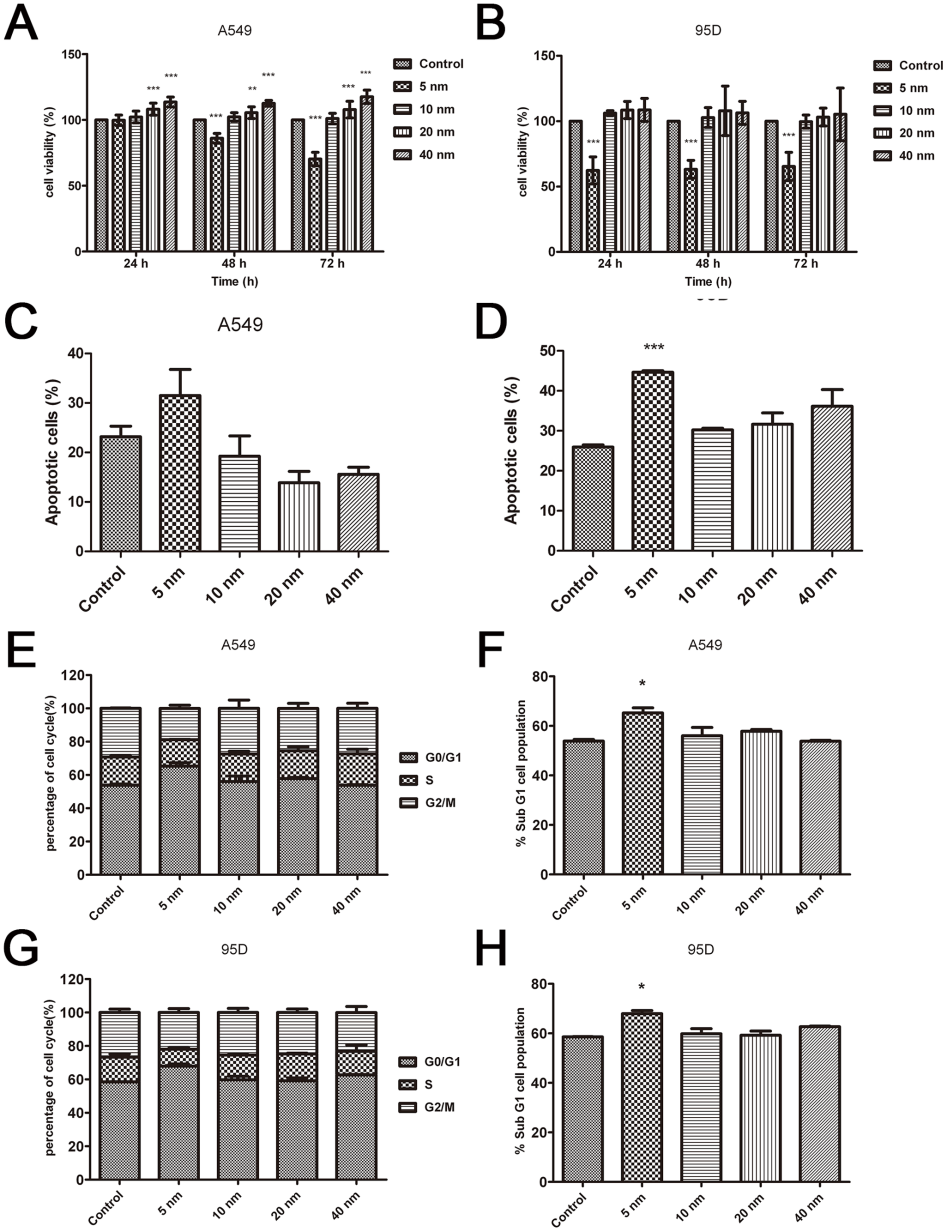
Comparison of the cytotoxicity of the A549 and 95D cell lines exposed to the Au-NPs. The cell lines A549 (A) and 95D (B) in the logarithmic growth phase were exposed to Au-NPs for 24 h, 48 h, and 72 h. Cell viability was calculated as the percentage of the viable cells compared to the untreated controls. Each result represents the mean viability ± standard deviation (SD) of three independent experiments. Apoptosis (C–D) and cell cycle (E–H) analyses of A549 and 95D cells treated with differently sized Au-NPs. Error bars indicate SD values from four independent experiments. *P<0.05, **P<0.01, ***P<0.001.

Next, we sought to determine the effect of these Au-NPs on the proliferation of two different lung cancer cell lines, A549 and 95D cells. The cytotoxic effect on the four Au-NPs with different diameters was tested at the same concentration. Our findings demonstrated that 5-nm Au-NPs play a pivotal role in inhibiting the proliferation of both A549 and 95D cells at 48 h and 72 h (Figures 3A and B). Au-NPs with diameters of 20 nm and 40 nm exhibited notable efficacy from 24 h onwards in promoting the proliferation of A549 cells, whereas no promotion was observed in 95D cells (Figures 3A and B). Au-NPs with a diameter of 10 nm had no effect on the proliferation of both A549 and 95D cells. Inhibiting the cell proliferation, increasing cell apoptosis, and arresting cells in the G0/G1 cycle manifested the cytotoxicity of 5-nm Au-NPs. There was no significant difference (P>0.05) in apoptosis and cell cycle distribution for the 10-nm, 20-nm, and 40-nm AuNP-treated groups (Figures 3C–H). These results indicated that small Au-NPs may be cytotoxic and that the diameter is not the only factor influencing cell proliferation and the interaction between Au-NPs, which is a very complex process that warrants further investigation.

### Invasion Assay

**Figure 4:**
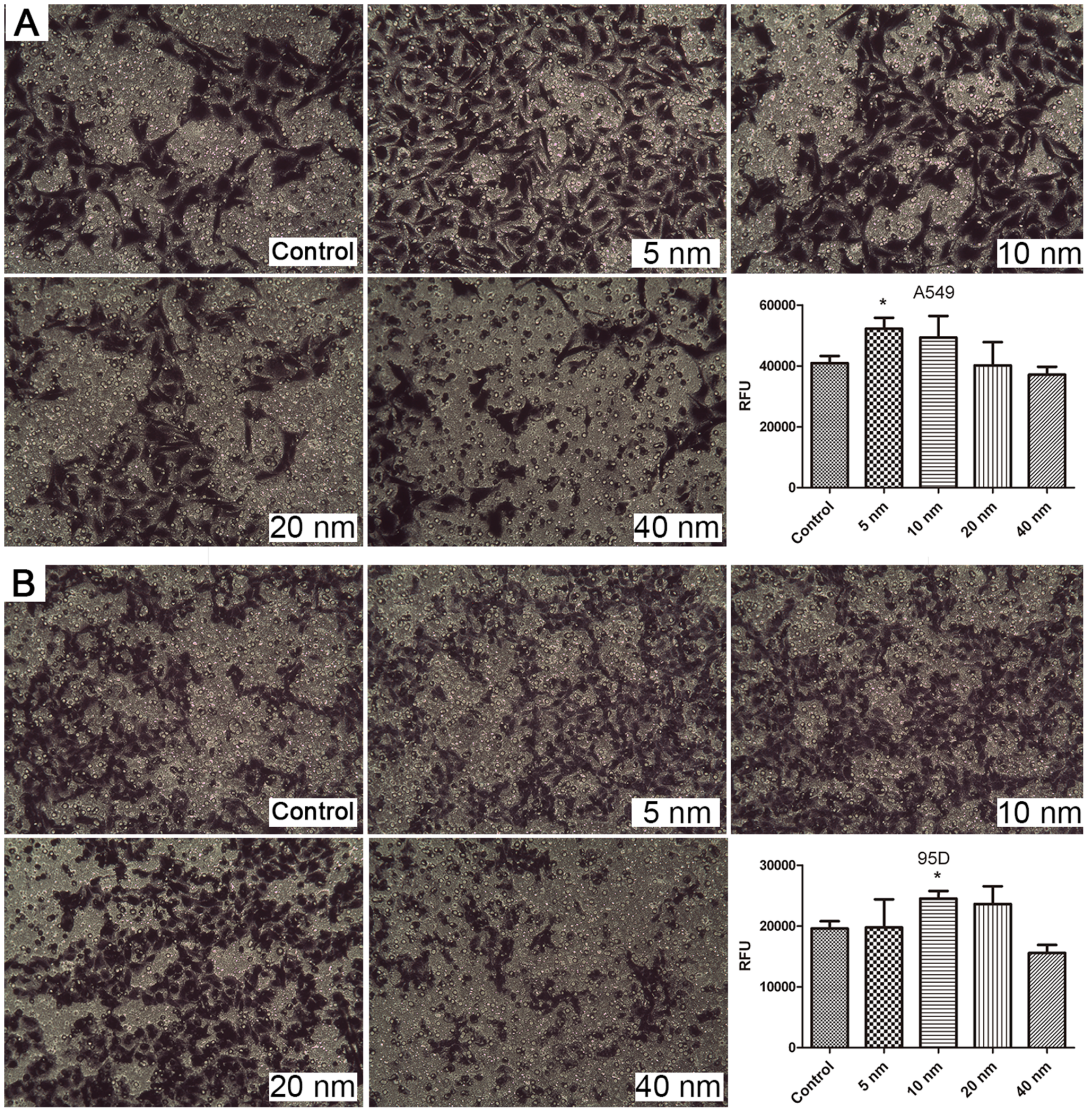
Optical images and fluorescence measurements of cell invasion on Au-NP treatment. The images represent A549 cells (A) and 95D cells (B) that have crossed the pores of the Matrigel invasion chamber and corresponding fluorescence intensity results. *P<0.05. Error bars indicate the SD values from three independent experiments.

A basement membrane model was used for evaluating the invasion activity of cells. The results are presented in Figure 4. Our results indicated that cell invasion was promoted significantly after treatment of A549 with 5-nm Au-NPs and of 95D cells with 10-nm Au-NPs (P<0.05). In contrast, the cells internalized with 20-nm or 40-nm Au-NPs were not significantly affected by invasion activity (Figure 4). These results clearly suggested that the invasion effects were particle size- and cell type-dependent.

### Effects of Au-NPs on the Expression of MMP9 and ICAM-1 in Lung Cancer Cells

**Figure 5:**
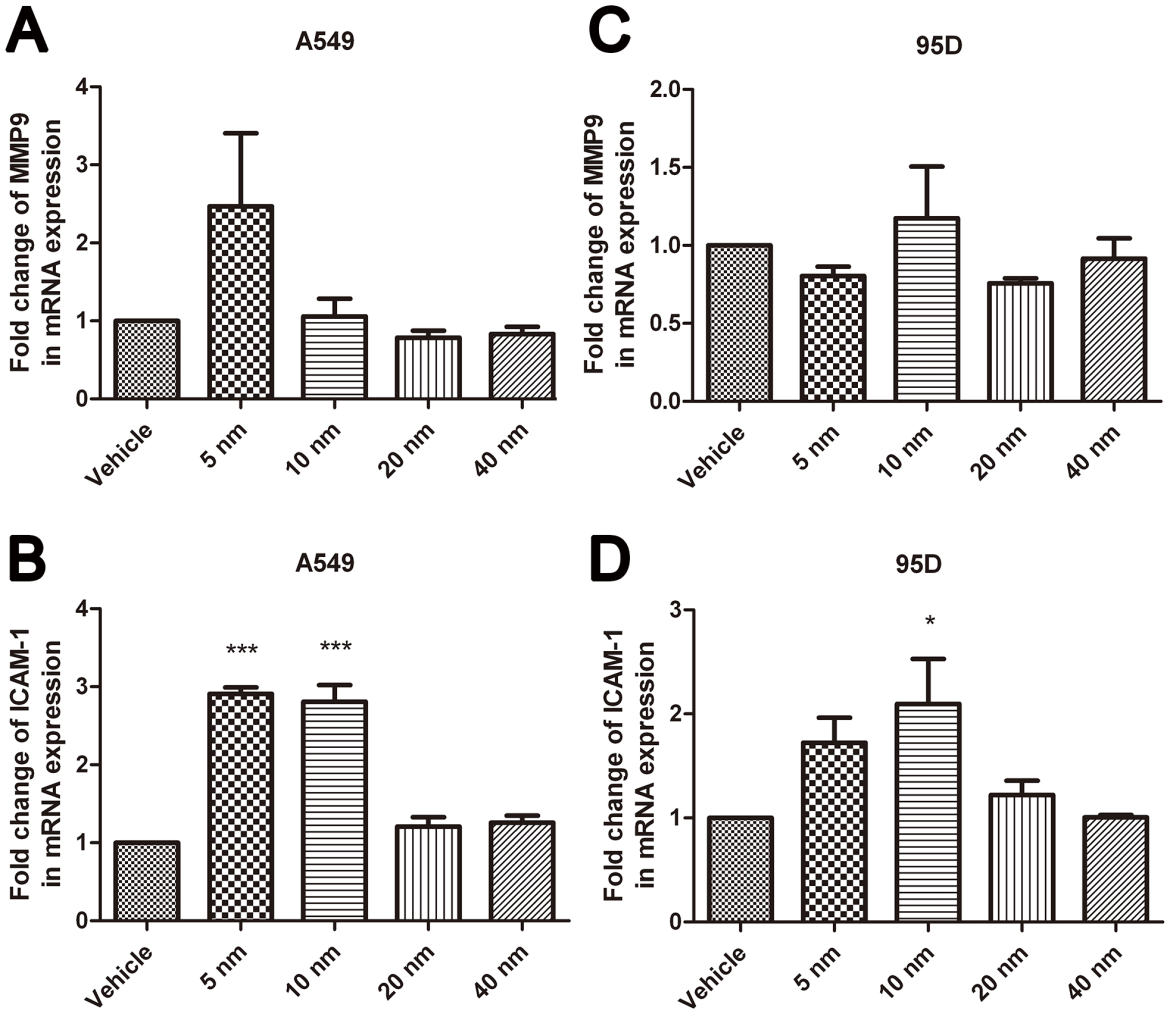
Fold changes in mRNA expression tested by qRT-PCR in A549 and 95D cells treated with differently sized nanoparticles. Results of qRT-PCR for (A–B) A549 and (C–D) 95D cells after 48-h incubation with 5-nm, 10-nm, 20-nm, or 40-nm Au-NPs. *P<0.05, ***P<0.001 vs. control. Error bars indicate the SD values from three independent experiments.

**Figure 6:**
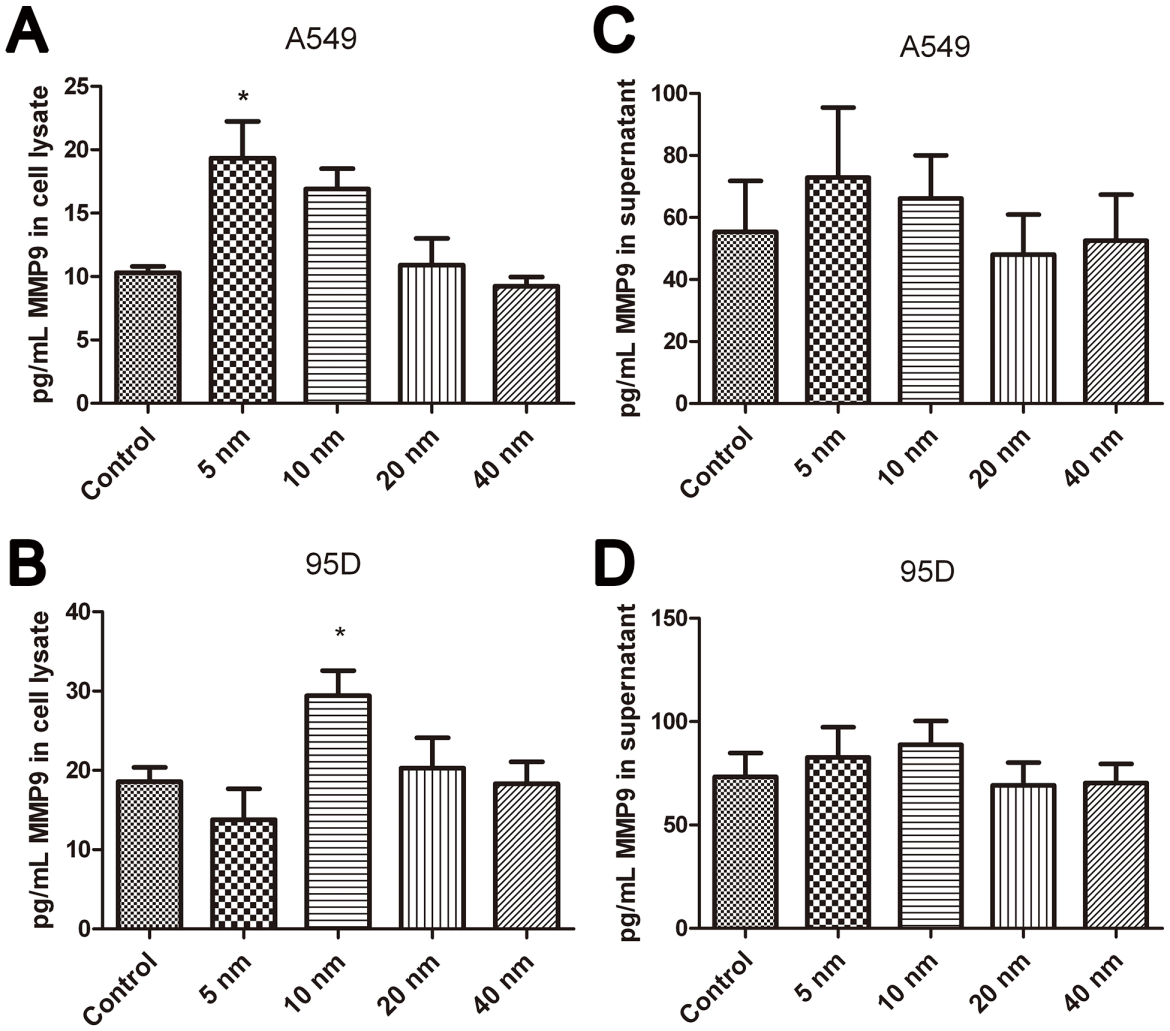
Expression levels of MMP9 in A549 and 95D cells after Au-NP treatment for 48 h. Quantification of the expression of MMP9 in the cell lysate (A–B) and the supernatant (C–D) of A549 and 95D cells, respectively, was conducted by using the Luminex technology. *P<0.05. Error bars indicate the SD values from three independent experiments.

To gain a deeper understanding of this phenomenon, qRT-PCR was performed to measure the mRNA levels of MMP9 and ICAM-1 after treatment with Au-NPs. The results showed that 5-nm and 10-nm Au-NPs notably facilitate the mRNA expression of ICAM-1 in both cell lines (Figures 5B and D, P<0.05). The mRNA expression of MMP9 increased in 5-nm Au-NPs–treated A549 cells and 10-nm Au-NPs–treated 95D cells but the differences were not statistically significant (Figure 5A and C). We then performed a Luminex-based experiment in the presence of MMP9 magnetic beads to quantify the protein expression of MMP9 both in the cell lysate and culture supernatant. Our results illustrated that treatment with 5-nm Au-NPs markedly increased the levels of MMP9 in the lysate of A549 cells, while MMP9 was notably upregulated in the lysate of 95D cells by treatment with 10-nm Au-NPs (Figures 6 A–B). The comparisons with the level of MMP9 in the supernatant revealed that there was no significant difference (P>0.05) between the Au-NP–treated and Au-NP–untreated A549 and 95D (Figures 6 C–D).

**Figure 7:**
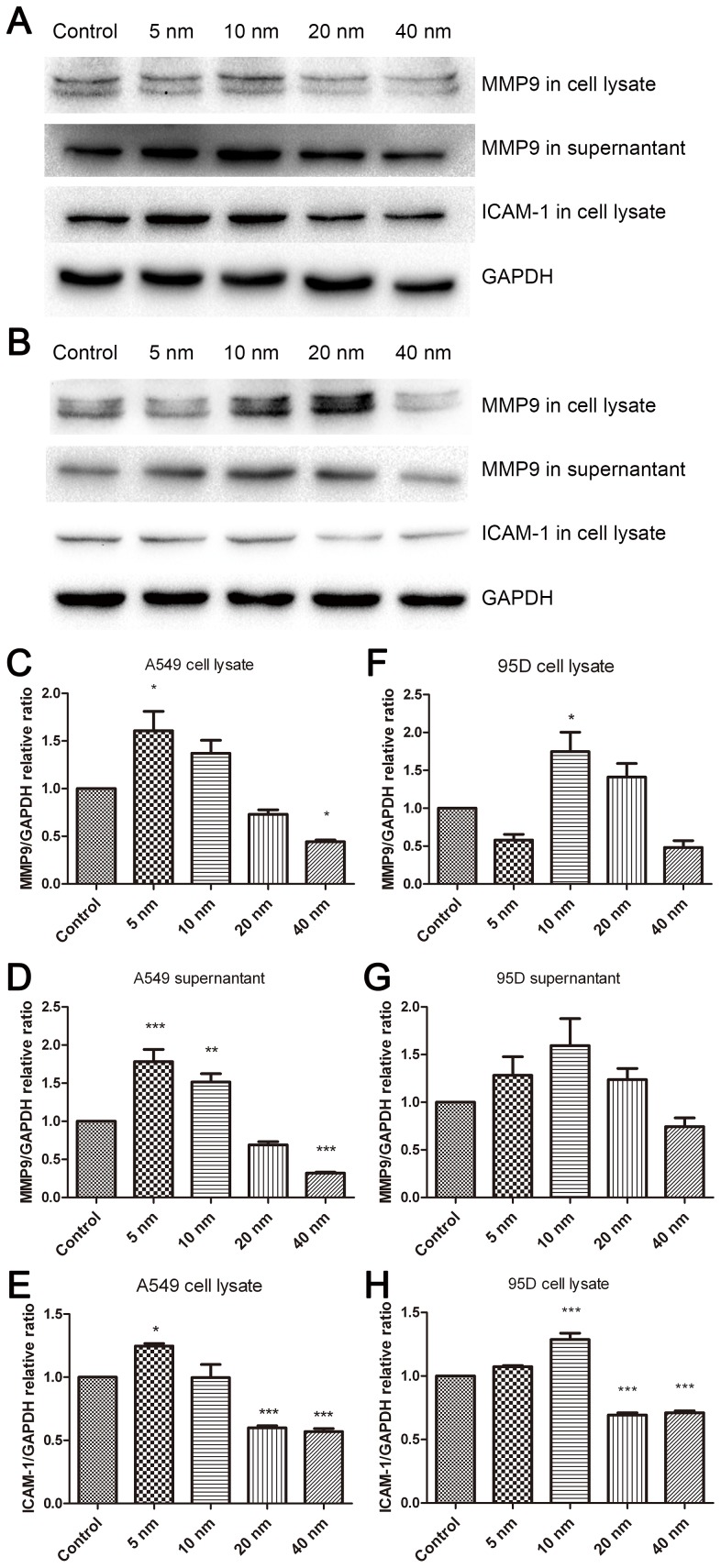
Western blot analysis of the expression of MMP9 and ICAM-1 in A549 and 95D cells. Representative results showing the effect of Au-NPs treatment on the expression of MMP9 and ICAM-1 in A549 (A) and 95D (B) cells. Relative band density of MMP9 and ICAM-1 to the mean value of the control group (C–H). Values are expressed as relative intensity normalized to GAPDH intensity and are given as mean ± SD from three independent experiments. *P<0.05, **P<0.01, ***P<0.001 vs. control.

Furthermore, we investigated the protein expression using western blotting. The results showed that MMP9 and ICAM-1 were upregulated in the lysates of A549 and 95D cells by treatment with 5-nm and 10-nm Au-NPs, respectively (Figure 7); in contrast, MMP9 and ICAM-1 were downregulated by treatment with 40-nm Au-NPs, suggesting that Au-NPs particle size plays an important role in the regulation of these proteins. These results provided the evidence that MMP9 and ICAM-1, key modulators of cell invasion, could be regulated by Au-NPs.

## Discussion

The low production cost and relative ease to synthesize Au-NPs make them feasible for future biomedical applications. However, with the transition of Au-NPs from the benchtop to the clinic, much more knowledge is required about the fundamental interactions between nanoscale materials and biological systems. The biosafety and biocompatibility of Au-NPs are vital concerns that should be demonstrated before such materials are applied to biological systems. Similar to many other reports [24], [25], in this study, Au-NPs were easily taken up by A549 and 95D cells via nonspecific endocytosis and localized within cytoplasmic vesicles. In this study, we demonstrated that small Au-NPs with a diameter of 5 nm exhibit high efficacy in inhibiting proliferation, promoting apoptosis, and arresting the cell cycle at the G0/G1 phase in two lung cancer cell lines. In contrast, no obvious cytotoxicity was observed in 10 nm, 20 nm, and 40 nm Au-NP–treated cells, which is in agreement with the results of other research groups. Previous studies showed that surface size, and not surface charge, plays a large role in the therapeutic effect of Au-NPs [26]. Generally, Au-NPs for drug delivery and photothermal therapy applications have dimensions larger than 10 nm, which is sufficient to decrease the surface reactivity and thus make gold cores benign. A size larger than 6–8 nm is also optimal for precluding renal excretion and consequently diminishing the circulatory half-life [27]. Unexpectedly, our results showed that the proliferation of A549 cells was promoted after 24-h treatment with Au-NPs of 20 nm or 40 nm in diameter. This phenomenon was not observed in 95D cells. Moreover, 5-nm Au-NPs and 10-nm significantly promoted the invasion of A549 and 95D cells, respectively, while the invasive ability was not affected in the presence of particles with a size greater than 20 nm. These results support the view that Au-NPs do not universally target all cell types [28]. Coulter et al. found that the surviving fraction for Au-NPs–treated cells showed a strong dependence on the cell type compared with that of untreated cells in respect to radiosensitization potential [24].

In our study, the improved invasion ability was accompanied by a notable upregulation of ICAM-1 and MMP-9 expression. Invasion through the ECM is an important step in tumor metastasis. Cancer cells initiate invasion by adhering to and spreading along the blood vessel wall. Matrix metalloproteinases are endopeptidases that are able to degrade ECM components, which allows cancer cells to access the vasculature and lymphatic systems[29]–[31]. MMP-9 has attracted much attention for its ability to degrade type IV collagen, the basic component of the basement membrane [32]. Increased expression of MMP-9 in patients with non-small cell lung cancer has been reported [33], [34]; therefore, agents suppressing the expression of the MMPs could inhibit cancer cell migration and invasion [35]. ICAM-1 is a representative adhesion molecule involved in the interaction among tumor cells, the endothelium, and ECM. High expression of ICAM-1 in human lung cancer specimens was correlated with a greater risk of advanced cancers (stages III and IV). A549/ICAM-1 cells were shown to induce in vitro cell invasion and in vivo tumor metastasis [36]. Denissenko et al. observed that ICAM-1 downregulation at the mRNA and protein levels led to strong suppression of human breast cell invasion through a Matrigel matrix [37]. We found that 5-nm and 10-nm Au-NPs effectively promoted the expression of ICAM-1and MMP9 in A549 and 95D cells, respectively, which partially explained the enhanced action of the particles on cancer cell invasion.

Since the upregulation effects of Au-NPs on MMP-9 and ICAM-1 expression in A549 and 95D cell suggested that small particles might possess the ability to facilitate the invasion of lung cancer cells, further in vivo studies are required to confirm the mechanisms. In summary, treatment with 5-nm Au-NPs effectively inhibited cells proliferation and promoted apoptosis, but it also upregulated the expression of ICAM-1 and MMP9, as well as increased the invasion of A549 cells. In contrast, Mukherjee et al. reported that Au-NPs (5 nm in diameter) exhibited anti-angiogenic properties (i.e., inhibited the tumorigenic growth of new blood vessels) both in vitro and in vivo [38]. Because of the complexity of this phenomenon and contradictory conclusions on the size of nanoparticles, further investigations are necessary to verify the effects and determine the appropriate size of Au-NPs for alleviating lung cancer progression.

This study provides new insights on the influence of Au-NPs of different sizes on cancer cell invasion, which could be of a great value for the application of Au-NPs to novel therapies in lung cancer. Our research indicated that the biological function of unmodified Au-NPs depended strongly on the particle size and cell type and that the particle size should be selected carefully for biological applications. There are both opportunities and challenges ahead in developing Au-NPs with suitable diameters that will be able to deliver novel anti-tumor agents or with intrinsic therapeutic potential for clinical use. In any case, the path toward the development of a feasible nanomedicine is still long and material safety and long-term bioeffects should be carefully considered.

## Conclusions

In this study, we have demonstrated the effects of citrate-capped Au-NPs of different sizes (5 nm, 10 nm, 20 nm, 40 nm) on the cytotoxicity and invasion in lung cancer cells. Based on the results, the following conclusions were drawn: First, nanoparticle size is an essential variable affecting cell proliferation, apoptosis, cell cycle, and cell invasion. Small particles endocytosed into the cells could cause great cytotoxicity, whereas large particles have no significant cytotoxicity. Second, in addition to particle size, cell type is also an important factor affecting the interaction between the Au-NPs and cells. Third, small Au-NPs upregulate the expression of MMP9 and ICAM-1, which may be associated with the increased invasion activity of A549 and 95D cells. This study provides useful information on cell cytotoxicity and invasion, although the molecular mechanisms need further investigation.
